# Endoscopic Ultrasound-Guided Therapies in Pancreatic Neoplasms

**DOI:** 10.1155/2015/731049

**Published:** 2015-01-31

**Authors:** Dennis Yang, Christopher J. DiMaio

**Affiliations:** Division of Gastroenterology, Mount Sinai Medical Center, New York, NY 10029, USA

## Abstract

Endoscopic ultrasound (EUS) has evolved from being primarily a diagnostic modality into an interventional endoscopic tool for the management of both benign and malignant gastrointestinal illnesses. EUS-guided therapy has garnered particular interest as a minimally invasive approach for the treatment of pancreatic cancer, a disease often complicated by its aggressive course and poor survival. The potential advantage of an EUS-guided approach revolves around real-time imaging for targeted therapy of a difficult to reach organ. In this review, we focus on EUS-guided therapies for pancreatic neoplasms.

## 1. Introduction

Since its introduction over 30 years ago, endoscopic ultrasound (EUS) has evolved from being primarily a diagnostic tool into a therapeutic modality for various gastrointestinal diseases. This transition towards “interventional EUS” has been facilitated by the advancement of curvilinear-array endoscopes and peripheral devices [1, 2]. EUS-guided fine-needle aspiration (EUS-FNA) is a well-established minimally invasive procedure that has been increasingly used for the investigation and staging of pancreatic neoplasms due to its favorable performance and safety profile when compared with other extracorporeal tissue acquisition techniques [3]. Intuitively, the ability to accurately and safely introduce a needle into a deeply located retroperitoneal organ (i.e., pancreas) serves as a vehicle for localized treatment. This is the basis for EUS-guided fine-needle injection (EUS-FNI) for pancreatic neoplasms: a safe, minimally invasive approach, with real-time imaging to selectively access deeply located targets and provide localized therapy. EUS-guided therapy may provide an important adjunctive treatment in patients with locally advanced, unresectable disease, or for those who are poor surgical candidates. Furthermore, the ability of EUS to accurately localize pancreatic lesions for tumor-marking may help guide systemic therapies and minimally-invasive parenchymal-sparing pancreatic surgery in select cases. In this review, we focus on advances of EUS-guided therapeutic approaches for pancreatic neoplasms.

## 2. Pancreatic Adenocarcinoma

Pancreatic ductal adenocarcinoma has a poor prognosis and represents the fourth leading cause of cancer-associated death in the United States. The disease is often advanced and unresectable (80%) at the time of diagnosis [4], limiting treatment options to systemic chemotherapy and/or radiation with considerable associated toxicities. EUS-FNI is a safe approach for the targeted delivery of therapeutic agents, which potentially facilitates direct intratumor therapy and possibly augments the effects of other established local and systemic therapies.  Table 1 summarizes human studies on EUS-guided therapies for pancreatic adenocarcinoma.

### 2.1. EUS-Guided Immunological Therapy

#### 2.1.1. Allogenic Mixed Lymphocyte Culture (Cytoimplant)

In 2000, Chang and colleagues investigated the feasibility and safety of immunotherapy by EUS-FNI in patients with advanced pancreatic adenocarcinoma [5]. In this phase I clinical trial, an allogeneic mixed lymphocyte culture (cytoimplant) was injected into the pancreatic tumor (*n* = 8) under EUS-guidance in a dose-escalating manner. The authors theorized that the injected cytoimplant would potentially stimulate local immune effector cells and release of cytokines resulting in an immune-mediated tumor regression. There were two partial responses (>50% tumor reduction on imaging), one minor response (<50% tumor reduction), three patients with no changes, and two with progressive disease. Overall, median survival was 13.2 months with no procedure-related adverse events. In spite of this early data supporting the feasibility and safety of EUS-guided cytoimplant delivery, there have since been no additional studies with this agent.

#### 2.1.2. Immature Dendritic Cells

Dendritic cells (DCs) are potent antigen-presenting cells capable of stimulating naïve T lymphocytes to develop into tumor-specific cytolytic cells. EUS-guided FNI allows the intratumoral injection of immature DCs. In principle, the localized DCs are then able to acquire and process tumor antigens in situ, migrate to regional lymphoid organs, and subsequently activate a tumor-specific immunological cascade. Akiyama et al. reported an 82% tumor growth inhibition of pancreatic cancer in hamsters with dendritic-based immunotherapy [6]. Later, Irisawa and colleagues investigated the effects of EUS-FNI of immature DCs in 7 patients with advanced pancreatic cancer refractory to gemcitabine [7]. Five of the 7 patients underwent irradiation prior to DC injection to produce tumor-associated antigens for DC cross-presentation. Three patients (43%) had a mixed response (regression of main pancreatic tumor but stable/progression of other lesions) and overall median survival was 9.9 months. There were no procedure-related adverse events. Subsequently, Hirooka et al. [8] investigated the effect of combination chemotherapy and DC immunotherapy for the treatment of locally advanced pancreatic cancer. The authors used OK432 as a maturation/activation agent of DCs to further enhance their activity on T-cell induction. Anti-CD3 monoclonal antibody (CD3-LAKs) was also administered to stimulate lymphokine-activated killer cells in the target tissue. Out of the five patients in this study, 1 had partial remission and two patients exhibited stable disease for more than 6 months. The median survival time was 15.9 months and there were no serious complications.

### 2.2. EUS-Guided Molecular Biological Therapy

#### 2.2.1. Oncolytic Virus Therapy (ONYX-015)

Advances in molecular biology and genetics have led to the emergence of cancer biotherapy with genetically engineered tumor-targeted microorganisms [9]. In 2003, Hecht and colleagues investigated the effects of EUS-FNI of ONYX-015 in unresectable pancreatic cancer [10]. ONYX-015 is a genetically engineered adenovirus with an E1B-55 kD gene-deletion which allows it to preferentially replicate in and kill malignant cells. There were no objective responses with ONYX-015 administration alone at day 35 (8 sessions over 8 weeks). When combination therapy with virus and gemcitabine was used in the final four treatments, partial regression of >50% was seen in 2/21 (10%), stable disease in 8, and progressive disease in 11 patients. Median survival time was 7.5 months. There were two patients with duodenal perforations and two with sepsis prior to adopting a transgastric EUS-FNI approach and prophylactic antibiotics as part of the protocol.

#### 2.2.2. Selective Delivery of Tumor Necrosis Factor-*α* (TNF-*α*)

Tumor necrosis factor-*α* has been recognized for its oncolytic potential through its effects on tumor vasculature and cytotoxicity [11]. TNFerade is an adenoviral vector containing a radiation-inducible Egr-1 promoter gene region upstream of the human TNF-*α* gene that, in theory, may selectively induce the antitumor activity of TNF-*α* in a specific target while minimizing its systemic toxicity. In 2012, Hecht et al. evaluated the effects of TNFerade combined with chemoradiotherapy on locally advanced pancreatic cancer in a phase I/II study [12]. TNFerade was administered via EUS-guidance (*n* = 27) or percutaneously (*n* = 23) once a week for 5 weeks together with radiation and 5-fluorouracil daily. Maximum tolerated dose was set at 4 × 10^11^ particle units/2 mL after 3/9 patients developed dose-limiting toxicity in the 1 × 10^12^ cohort. Complete response was seen in 1 (2%) patient and partial response in 3 (6%) patients, whereas 12 (24%) and 19 (38%) patients had stable and progressive disease, respectively. Overall median survival was 297 days, with the best mean survival (332 days) seen in the 4 × 10^11^ cohort. The method of TNFerade administration (EUS versus percutaneous route) did not influence overall survival. More recently, a randomized phase III multicenter trial compared 5-FU chemoradiation therapy with or without TNFerade [13]. The study was stopped early as the TNFerade with chemoradiation therapy arm was associated with inferior progression-free survival and higher toxicity when compared to standard of care alone. Thus, given these disappointing results, there are currently no active clinical trials evaluating this agent for the treatment of pancreatic cancer.

#### 2.2.3. Delivery of Intratumoral Paclitaxel

The application of EUS-guided injection techniques for the delivery of intratumoral chemotherapeutic agents is an exciting prospect, particularly for patients with locally advanced, unresectable disease. Thus far, experience with this concept has been limited to safety and feasibility studies in animal models. Matthes and colleagues [14] reported the successful delivery of an intralesional injectable formulation of paclitaxel (OncoGel, MacroMed Inc., Sandy, UT, USA) in a porcine pancreas model. The OncoGel (ReGel/paclitaxel) is composed of a polymer that, upon injection and in response to body temperature, transforms into a water-insoluble biodegradable hydrogel that releases paclitaxel into the target tissue for up to 6 weeks. Using an AVA-TEX threaded syringe (Cardinal Health, Dublin, OH) preloaded with OncoGel attached to a 22-gauge EUS needle, different volumes were injected transgastrically into the tail of the porcine pancreas (*n* = 8). Successful localized intrapancreatic collection of the OncoGel depot was seen on follow-up contrast-CT and on gross tissue inspection after euthanasia. There were three accidental extrapancreatic injections to sites close in location to and in addition to the main depot in the porcine pancreatic tail. Despite these incidences, all animals tolerated the procedure without clinical sequelae. Following this study, the same group investigated the feasibility of EUS-guided delivery of irinotecan-loaded microspheres into the swine pancreas [15]. Using a 19-gauge EUS needle and via a transgastric approach, a range of doses of irinotecan with LC beads were injected into the pancreatic tail parenchyma. Histopathologic examination in 10/12 pigs confirmed the presence of a foreign-body, giant-cell reaction, and granulation tissue. The dose of the irinotecan did not correlate with the grade of the reaction or the size of the depot on CT imaging.

Overall, EUS appears to be ideally suited for the guided-administration of biologic agents into the pancreas. Initial studies of EUS-FNI of antitumor agents have been promising and suggest that it is both safe and feasible. However, large clinical trials are needed to confirm these preliminary findings before these investigational therapies can be implemented in clinical practice.

### 2.3. EUS-Guided Physiochemical Therapy

#### 2.3.1. EUS-Guided Brachytherapy

Brachytherapy is a form of radiation therapy that allows localized high doses of targeted radiation while reducing radiation exposure to surrounding normal tissue. While brachytherapy has been well established for the treatment of many cancers, its application in pancreatic cancer is still in its infancy. The feasibility of EUS-guided interstitial brachytherapy of the pancreas in an animal model was first reported by Sun and colleagues in 2005 [16]. Iodine I radioactive seeds were inserted into the lumen of the tip of a modified EUS 22-gauge needle and subsequently implanted into the normal porcine pancreas via a transgastric approach. There was no seeds migration or procedure-related complications. Histopathologic examination confirmed a localized zone of necrosis and fibrosis in the target area in each specimen. Subsequently, the same group reported their results on EUS-guided interstitial brachytherapy in 15 patients with unresectable pancreatic cancer [17]. A mean number of 22 iodine-125 radioactive seeds were implanted in each patient through a 19-gauge needle. Grade III hematologic toxicity and mild pancreatitis occurred in 3 (20%) patients. Four patients showed partial response, 3 minor response, 5 stable disease, and 3 disease progression. Clinical benefit (measured by reduction in pain level or improvement in Karnofsky performance status (KPS) score) was noted in 5 (30%) patients. Overall median survival was 10.6 months. Later, Jin et al. investigated the combined effect of EUS-guided brachytherapy with gemcitabine-based 5-FU for advanced pancreatic cancer [18]. Most patients (19/22) underwent one implantation session (median number of 10 iodine-125 seeds/session). One week following initial implantation, patients started a 5 day intravenous schedule of gemcitabine/5-FU. The regimen was repeated every 4 weeks up to 6 cycles if tolerated. Partial response was reported in 3 (13.6%) patients, stable disease in 10 (45.5%), and disease progression in 9 (40.9%). The VAS pain score and KPS score were both significantly lower at 1 month following therapy. The estimated median survival time was 9 months based on their Kaplan-Meier analysis. More recently, this same group presented their long-term results on EUS-guided brachytherapy combined with gemcitabine in 100 patients with unresectable pancreatic cancer [19]. Median number of seeds implanted was 24.1 per patient. Following implantation, patients received gemcitabine every 4 weeks up to 6 cycles if tolerated, except in 15 patients who did not accept the chemotherapy. Mean follow-up time was 7.8 months and estimated one- and two-year survival rates were 21% and 4%, respectively. The mean VAS pain score decreased significantly at 1 month. Thus, while EUS-guided interstitial brachytherapy was associated with improvement in quality of life as measured by pain score reduction, there was no evidence of survival benefit.

The main barrier to widespread use of EUS-guided brachytherapy, however, remains the limited availability of this technique. In addition, the special training, handling, precautions, and regulations needed to handle and delivery radioactive devices may be prohibitive to performing this technique in most endoscopy units. Joint collaboration with colleagues in radiation oncology is needed.

#### 2.3.2. EUS-Guided Fiducial Implantation

Fiducials are radiopaque markers that can be implanted in or near the tumor target for precise target localization and are key for image-guided radiation therapy (IGRT). The feasibility and safety of EUS-guided fiducial marker placement in pancreatic cancer was initially described by Pishvaian et al. in 2006 [20]. Later, a single-center prospective study reported the successful placement of gold fiducials through a 19-gauge needle under EUS-guidance in 50 out of 53 patients with locally advanced unresectable cancer [21]. With the introduction of smaller fiducials, there have been reports on successful EUS-guided fiducial placement for mediastinal and upper GI malignancies through a 22-gauge needle [22]. More recently, Draganov and colleagues evaluated a dedicated EUS-guided multifiducial delivery system in an animal model and reported a fiducial deployment success rate of 95.6% without adverse events [23].

**Figure 1:**
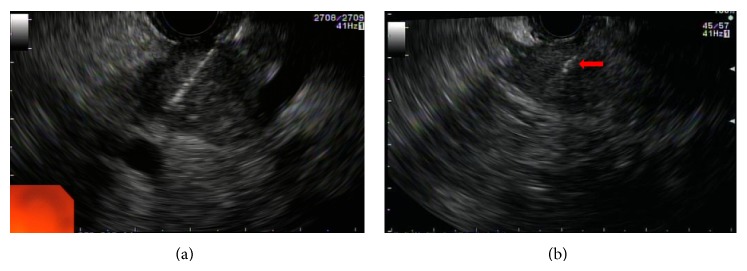
A 22-gauge needle was inserted into a 25 × 18 mm malignant hypoechoic mass lesion in the pancreas body for fiducial placement (a). Postimplantation EUS confirming placement of a 10 mm × 0.35 mm Visicoil fiducial maker (Core Oncology, Santa Barbara, CA, USA) within the lesion (*arrow*) (b).

In summary, the technical success of EUS-guided fiducial placement in pancreatic cancer is high, ranging from 85 to 100% [24], with minimal complications reported (Figure 1). Thus far, studies on EUS-guided fiducial placement have been largely of proof-of-concept, and outcome measures, such as decreased radiotoxicity and improved survival, are currently lacking. Further studies are needed in order to establish the role and impact of fiducial implantation in the management of pancreatic adenocarcinoma.

#### 2.3.3. EUS-Guided Delivery of Ablative Energy

There have been multiple studies evaluating the technical feasibility and efficacy of EUS as a tool for the delivery of targeted ablative energy. The results of these EUS-guided ablation techniques on porcine models are summarized in Table 2.

Radiofrequency ablation (RFA) relies on the generation of high-frequency alternating electromagnetic energy resulting in thermal injury to the target tissue [25]. The role of RFA in the pancreas has been limited by its restricted accessibility by a percutaneous approach. Thus, EUS may be a suitable alternate vehicle for RFA therapy. EUS-guided RFA in the pancreas was initially explored in a porcine model by Goldberg and colleagues in 1999 [26]. Under EUS-guidance, a 19-gauge needle electrode was passed transgastrically to the pancreatic tail to deliver the RF current. Successful coagulation necrosis of the targeted areas was achieved in all the animals and confirmed by both radiologic and pathologic examination. Mild hyperlipasemia, focal pancreatitis, and subsequent pancreatic fluid collections were reported in 1 pig. More recently, Kim et al. [27] reported the efficacy and safety of EUS-RFA by puncturing the porcine body and tail of the pancreas with an 18-gauge RFA electrode via a transgastric approach. EUS-RFA resulted in well-demarcated ablation lesions in all pathology specimens and no procedure-associated adverse events. Advances in RFA probes have led to development of new devices. In 2008, Carrara et al. [28] reported their experience with a hybrid cryotherm probe (CTP) that combined bipolar RF and cryogenic cooling in a porcine model. The authors successfully performed RF in the normal pancreatic body (*n* = 14), noting a positive correlation between ablation zone and duration of treatment. Reported adverse events included 1 case of necrotic pancreatitis with peritonitis, 1 histologically proven pancreatitis without clinical symptoms, 1 thermal injury to the gastric wall, and four adhesions between the pancreas and the gut. In addition, there has been one report on EUS-guided RFA of the proximal pancreas in a porcine model [29]. In this study, Gaidhane and colleagues used a 19-gauge needle to puncture the head of the pancreas (*n* = 5) via a transduodenal approach under EUS-guidance. A pilot monopolar RFA probe was subsequently advanced through the needle and RF was performed. Only 1/5 animals showed moderate level of pancreatitis (coagulative necrosis) with 20% involvement of the proximal pancreatic tissue. The lack of effectiveness of EUS-RFA in this study was attributed to technical limitations of the device and poor visualization and access of the proximal porcine pancreas.

Photodynamic therapy (PDT) is based on the ability of photosensitizers to generate cytotoxic oxygen species in the target tissue upon exposure to light of an appropriate wavelength [30, 31]. The feasibility and safety of EUS-guided PDT was initially studied in 2004 by Chan and colleagues in a porcine model [32]. After injecting the photosensitizer porfimer sodium, the investigators introduced a 19-gauge needle under EUS guidance followed by the insertion of a quartz optical fiber to deliver the PDT. There were localized areas of coagulation necrosis with low-dose PDT in the normal pancreas (*n* = 3; 9 applications) without immediate or delayed complications. Subsequently, Yusuf et al. [33] investigated the effects of EUS-guided PDT through a 19-gauge needle in the normal porcine pancreas with verteporfin, an agent that has been associated with less photosensitivity than porfimer sodium. The pigs were randomly divided into three groups and exposed to laser light for 10, 15, or 20 minutes. The size of the lesion from PDT corresponded to the length of exposure to the laser light and there were no complications reported.

Neodymium-doped yttrium aluminum garnet (Nd:YAG) laser is a solid-state laser that emits light at mid-infrared wavelengths of varying pulse duration and energy in order to penetrate tissue and induce phototoxicity and necrosis [34]. The potential advantage of this modality is the attributed precision of laser-induced tissue necrosis. di Matteo and colleagues reported EUS-guided Nd:YAG laser ablation in a porcine model in 2010 [35]. Nd:YAG laser ablation was performed with an optical fiber introduced through a 19-gauge needle inserted into the normal pancreas (*n* = 8) under EUS-guidance. A well-demarcated ablation area was seen on histopathologic examination and there were no major complications. More recently, the same group [36] investigated optimal Nd:YAG laser settings by evaluating ablation volume and central carbonization volume, a measure presumed to reflect unintended surrounding thermal injury. Their results demonstrated that ablation volume increased with laser output power up to 10 W, but those subsequent increments in output power to 20 W were associated with larger carbonization volumes without increases in the ablation volume. The authors concluded that ablation and carbonization volumes could be used to define effective yet safe therapeutic Nd:YAG laser settings [36].

High-intensity focused ultrasound (HIFU) is an evolving technology that delivers ultrasound energy to the target tissue resulting in an elevation in temperature leading to tissue denaturation [37]. Previous studies have evaluated HIFU for pain management in pancreatic cancer [38, 39]. Subsequently, Hwang and colleagues have evaluated the safety and feasibility of extracorporeal ablation of HIFU in a porcine pancreas model [40]. The authors treated the animals (*n* = 12) with extracorporeal HIFU at different acoustic treatment energies. The degree of ablation identified on histology correlated with the treatment energy. However, at effective treatment energy, thermal injury to the abdominal wall and gastric ulcers were also reported in the animal model. More recently, other studies have suggested that HIFU may also act synergistically by augmenting target drug delivery and promote an antitumor immunological response [41, 42].

EUS-guided delivery of ablative energy is a promising treatment modality. The development of novel devices dedicated to this purpose is necessary to allow widespread implementation of this technique. Questions still remain regarding the safety of these techniques and the overall impact on disease status. However, given the use of such techniques in malignant disease in other organs (e.g., liver), there is a strong interest in the use of EUS-guided ablation as an adjunct to other accepted modalities.

## 3. Pancreatic Neuroendocrine Tumors

Pancreatic neuroendocrine tumors (pNET) account for only a small percentage (3–5%) of pancreatic neoplasms and, in general, carry a better prognosis than pancreatic adenocarcinoma [43]. While surgical resection is the mainstay therapy, this may not be suitable for patients with metastatic disease or those with prohibitive medical comorbidities. EUS-guided therapy is becoming a promising treatment modality for pNETs.

### 3.1. EUS-Guided Ethanol Ablation

**Figure 2:**
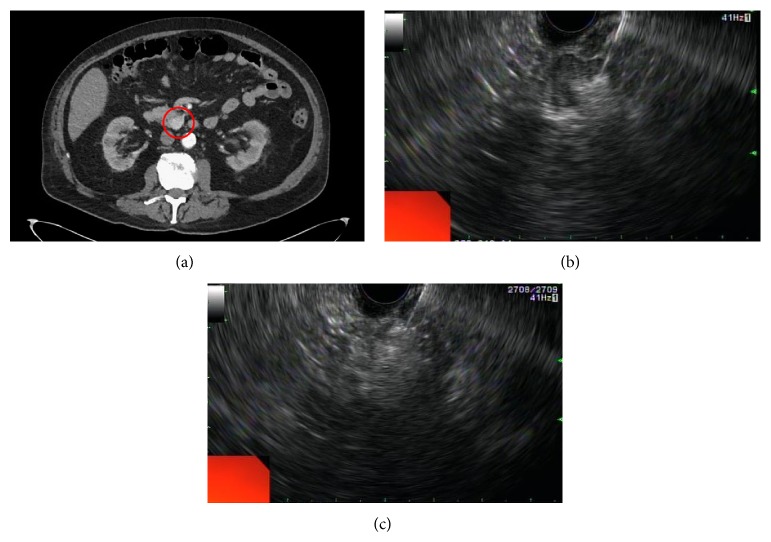
Computed tomography (CT) of the abdomen/pelvis reveals a 22 × 13 mm enhancing mass lesion (circle) located in the pancreatic head (a). EUS-guided ethanol ablation is performed with the 22-gauge needle by injecting 98% alcohol in 0.01 mL to 0.1 mL aliquots (b). Repeated injections are performed until a hyperechoic blush is seen expanding in the tumor (c).

Ethanol has been commonly employed as an ablative agent in renal and hepatic cystic lesions given its ability to cause cell membrane lysis and vascular occlusion [2]. Recently, this agent has been evaluated for the treatment of pNET. In 2006, Jürgensen et al. reported the successful treatment of an insulinoma by EUS-guided ethanol ablation in a symptomatic nonsurgical candidate 78-year-old woman [44]. After treatment, the patient did not endorse any further hypoglycemic episodes or evidence of the lesion on follow-up EUS. Similarly, two other case reports have also alluded to the successful treatment of symptomatic sporadic insulinomas with EUS-guided ethanol ablation [45, 46]. More recently, Levy and colleagues retrospectively reviewed and reported their experience on US-guided ethanol ablation of insulinoma in eight patients [47]. Five patients underwent EUS-guided ethanol ablation whereas the remaining three underwent intraoperative ultrasound- (IOUS-) guided ablation. For the EUS group, a 22- or 25-gauge needle was advanced into but not through the tumor and small aliquots (0.01–0.1 mL) of ethanol were injected at a time. This process was repeated until a hyperechoic blush was seen expanding within the tumor (Figure 2). There were no complications during or after the EUS-guided procedure. On the other hand, IOUS-guided therapy was associated with a minor peritumoral bleeding (*n* = 1), pseudocyst (*n* = 1), and rehospitalization in one patient due to pancreatitis. Overall, hypoglycemia-related symptoms resolved completely following EUS-guided treatment and improved in those who underwent the IOUS approach.

EUS-guided ethanol ablation is increasingly being recognized as a viable alternative treatment modality in patients with symptomatic pNETs. It should be stressed that the goal of this treatment is biologic control of hormone overproduction, as opposed to oncologic cure. As such, this treatment modality may be best reserved for those patients who are not surgical candidates and/or cannot tolerate medical management.

### 3.2. EUS-Guided pNET Localization

Precise localization of small pNETs at the time of surgery can often be challenging. Preoperative planning and lesion localization are crucial to ensure tumor-free surgical margins while sparing normal parenchyma. Hence, EUS-guided tumor localization and marking prior to surgical intervention has been explored. In 2002, Gress et al. reported a successful case of EUS-guided fine-needle tattooing (FNT) with diluted India Ink (Permark, Inc., Edison, NJ) for preoperative localization of an insulinoma in a patient prior to laparoscopic surgery [48]. More recently, Lennon et al. reviewed the feasibility, safety, and efficacy of EUS-FNT for tumor localization prior to laparoscopic distal pancreatic resection [49]. The authors used a 22-gauge EUS-FNA needle to inject 1–5 mL of sterile purified carbon particles (GI Spot; GI Supply, Camp Hill, PA) into the pancreatic parenchyma immediately adjacent (3–5 mm) to the lesion. GI spot was injected in increments until a hyperechoic blush was seen and continued as the needle was withdrawn, leaving an inked tract and a small subcapsular bleb of ink. The authors report that the tattoo was clearly seen at the time of surgery in all 13 patients who underwent EUS-FNT. Furthermore, all of these patients had negative final margins by pathology and none had positive frozen section on final evaluation.

The same group recently reported the efficacy of fiducial implantation for tumor localization in two consecutive patients with pNET prior to surgical resection [50]. Using a 22-gauge FNA needle backloaded with a Visicoil fiducial (Core Oncology, Santa Barbara, CA, USA), the authors placed the fiducials within the lesions (pNET at the uncinate/neck of the pancreas in both cases). The fiducials were identified successfully on intraoperative ultrasound and both patients had the lesions enucleated successfully. There were no periprocedural complications.

In summary, based on numerous small case series, EUS-guided tumor localization with FNT or fiducial placement appears to be both safe and feasible.

## 4. Pancreatic Cysts

Pancreatic cysts are increasingly being discovered incidentally, to some extent paralleling the increased utilization of cross-sectional imaging [51]. Their management can be challenging as imaging and sometimes even EUS-guided FNA are not always successful in differentiating the various types of cystic lesions [52, 53]. Surgical resection, the mainstay of therapy for those lesions with malignant potential, is often associated with substantial morbidity. Furthermore, the preoperative risk stratification of who may benefit the most from surgical resection relies on suboptimal diagnostic tests. Hence, there is a growing interest in pancreatic cyst ablation as an alternative modality for these lesions and EUS-FNI may represent an ideal vehicle to guide therapy under real-time imaging.

### 4.1. EUS-Guided Ethanol Ablation

**Figure 3:**
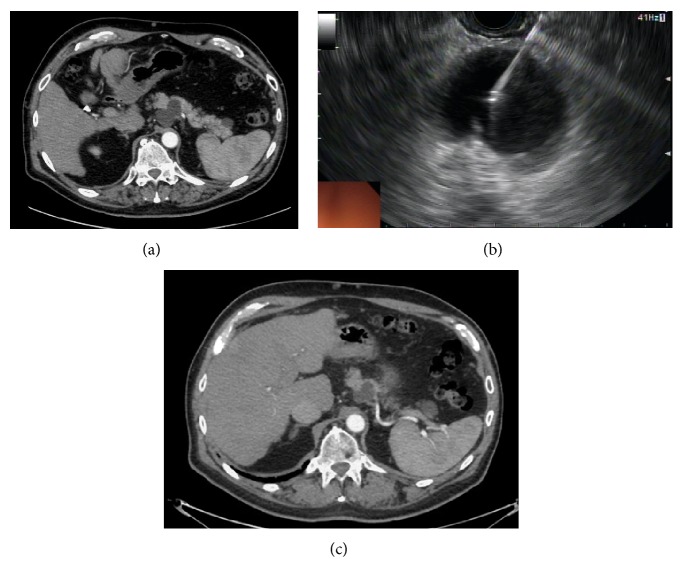
CT of the abdomen/pelvis reveals a 43 × 27 mm pancreas cyst in the body (a). EUS-guided ethanol ablation of pancreas cyst (b). Follow-up CT scan showing decrease in size (26 × 19 mm) of pancreas cyst after EUS-guided ethanol ablation (c).

The feasibility of EUS-guided ethanol injection in a normal porcine pancreas model was first reported by Aslanian et al. in 2005 [54]. Ethanol (either 50% or 98% ethanol) was injected with a 22-gauge needle into the pancreas (*n* = 8) under EUS guidance. The 50% ethanol injections led to localized ablation zones whereas those subjected to 98% ethanol revealed more extensive injury and unpredictable pancreatitis. Since this initial study in an animal model, there have been several studies evaluating the effects of EUS-guided ethanol ablation in pancreatic cystic lesions (Figure 3). These studies are summarized in Table 3. In 2005, Gan and colleagues published their series on ethanol lavage of pancreatic cysts in 25 patients [55]. Overall, ethanol lavage reduced tumor size without adverse effects in both short and long term follow-up. Cyst resolution was not influenced by the ethanol concentration administered (range 5–80%). In total, eight patients (35%) had complete resolution of their cysts on follow-up imaging.

A multicenter randomized controlled trial in 2009 investigated the effects of ethanol compared to saline lavage on pancreatic cystic lesions [56]. Twenty-five patients were treated with 80% ethanol lavage and 17 patients received saline lavage. Three months following initial lavage, all patients (*n* = 47) received an 80% ethanol lavage. Ethanol lavage resulted in greater size reduction of the pancreatic cystic tumors compared to saline lavage and complete cyst resolution was also achieved in 33.3% (6/18) of patients who received ethanol. Subsequently, another study [57] reported that pancreatic cyst size decreases significantly after 2 ethanol lavage sessions. In addition, complete cyst resolution was not accomplished after 1 session but noted in 38% (5/13) who underwent 2 lavages. Similarly, the relative durable response to ethanol ablation was reported by Dewitt et al. in a prospective cohort (*n* = 9) with a median follow-up of 26 months [58].

Based on the principle of EUS-guided ethanol ablation for pancreatic cystic neoplasms, Oh and colleagues [59] evaluated the feasibility and safety of combined EUS-guided ethanol lavage with paclitaxel injection (EUS-ELPI). EUS-guided ethanol lavage (99% ethanol) and paclitaxel injection were performed successfully in 13 out of 14 asymptomatic patients with pancreatic cysts. At a median follow-up of 9 months, complete cyst resolution was observed in 11 patients, and partial resolution in 2 patients. One patient experienced mild pancreatitis. Following this initial study, the same group [60] reported their experience on EUS-ELPI on 52 patients. Original cyst volume was the only factor predictive of complete cyst resolution, which was achieved in 29 patients (62%) at a median follow-up of 21.7 months. The positive yet preliminary findings from these authors warrant further studies to delineate the role of chemotherapeutic agents for the management of pancreas cyst tumors.

In aggregate, most of these studies suggest that pancreatic cyst ablation is safe and feasible. Nonetheless, except for a single randomized study, the majority of the published literature revolves around single-center case series. Furthermore, given the well-recognized limitations on pancreatic cyst diagnosis based on clinical criteria (i.e., cyst morphology, size, cytology, lab analysis, and tumor markers), in the absence of a surgical pathology specimen confirmation, it is difficult to determine the definite effect of EUS-guided ethanol ablation. In addition, issues arise also on how to survey these patients following therapy, as it remains unclear whether radiologic resolution equates with pathologic resolution. In the absence of large prospective, randomized studies with long-term follow-up comparing cyst ablation with radiographic surveillance, EUS-guided ethanol ablation should still be reserved for investigational protocols.

## 5. Conclusion

EUS has emerged as an interventional endoscopic tool for the management of pancreatic neoplasms. EUS-guided therapy is a promising minimally invasive approach that permits real-time imaging for the delivery of multiple therapeutic modalities, including various ablative techniques, antitumor agents, and local irradiation. The main advantage of this technique is the localized delivery of high concentrations of a therapeutic agent or ablative energy, with the potential for minimal systemic toxicity. Future research involving large prospective studies is necessary to better delineate the role of EUS-guided therapy in pancreatic neoplasms, with particular interest in those with locally advanced disease. In addition, the development of dedicated devices designed to work with current FNA needles is required.

## Figures and Tables

**Table 1:** EUS-guided therapy for pancreatic adenocarcinoma (human studies).

Technique	References	*N*	Outcome	Adverse events
Immunologic therapy				
Cytoimplant	Chang et al. 2000 [5]	8	2 partial response 1 minor response 3 stable disease 2 progressive disease	None
Immature DCs	Irisawa et al. 2007 [7]	7	3 mixed response^*^ 1 stable disease 3 progressive disease	None
Gemcitabine and DCs	Hirooka et al. 2009 [8]	5	1 partial response 2 stable disease 2 progressive disease	Leukopenia (*n* = 1) Anemia (*n* = 2) Nausea and constipation (*n* = 1)

Biologic therapy				
Oncolytic virus (ONYX-015)	Hecht et al. 2003 [10]	21	2 partial response 8 stable disease 12 progressive disease	Sepsis (*n* = 2) Duodenal perforation from EUS-FNI (*n* = 2) Leukopenia (*n* = 7) Anemia (*n* = 1)
TNFerade (EUS *n* = 27, percutaneous *n* = 23)	Hecht et al. 2012 [12]	50	1 complete response 3 partial response 4 minor response 12 stable disease 19 progressive disease	GI bleeding (*n* = 6), DVT (*n* = 6), PE (*n* = 2), abdominal pain (*n* = 9), pancreatitis (*n* = 2), cholangitis (*n* = 6) biliary obstruction (*n* = 8), coagulopathy (*n* = 3), nausea/vomiting (*n* = 4), hypotension (*n* = 2), intestinal ischemia (*n* = 1), cardiopulmonary events (*n* = 4), electrolyte abnormalities (*n* = 5), death unrelated to treatment (*n* = 2)

Physiochemical therapy				
EUS-guided interstitial brachytherapy	Sun et al. 2006 [17]	15	4 partial response 3 minor response 5 stable disease 3 progressive disease	Leukopenia (*n* = 4), anemia (*n* = 5), thrombocytopenia (*n* = 3), abnormal liver tests (*n* = 4), nausea/vomiting (*n* = 2), fever (*n* = 3), infection (*n* = 3), constipation (*n* = 2), diarrhea (*n* = 3)
	Jin et al. 2008 [18]	22	3 partial response 10 stable disease 9 disease progression	Seed translocated to liver 24 h after procedure (*n* = 1), fevers (*n* = 12), amylase elevation (*n* = 1)

^*^Regression of main pancreatic tumor but stable/progression of other lesions.

**Table 2:** EUS-guided delivery of ablative energy.

Technique	References	Model	*N*	Outcome	Adverse events
	Goldberg et al. 1999 [26]	Swine pancreas	13	RFA treatment effect (coagulation necrosis and/or fibrosis) seen on pathology on all specimens.	Gastric burns (*n* = 3) Intestinal serosa burn (*n* = 1) Pseudocyst (*n* = 1).
Radiofrequency ablation (RFA)	Kim et al. 2012 [27]	Swine pancreas	10	Ablated lesion with surrounding normal pancreatic parenchyma seen on all pathology specimens.	None.
	Gaidhane et al. 2012 [29]	Swine pancreas	5	RFA treatment effect (coagulative necrosis) in head of pancreas seen only in 1/5.	None.

RFA and cryothermal treatment	Carrara et al. 2008 [28]	Swine pancreas	14	RFA treatment effect (coagulation necrosis) in 12/14 animals.	Necrotizing pancreatitis (*n* = 1), pancreatitis without symptoms (*n* = 1), gastric burn (*n* = 1), adhesions between pancreas and gut (*n* = 4).

Photodynamic therapy (PDT)	Chan et al. 2004 [32]	Swine liver, pancreas, kidney, spleen	3	PDT treatment effect (coagulation necrosis) was 100% in the pancreas (9 applications).	Gross ecchymosis on surface of pancreas (*n* = 1).
	Yusuf et al. 2008 [33]	Swine pancreas	6	PDT treatment effect (focal fat necrosis) on gross pathology on 6/6 pigs.	Elevated serum amylase (*n* = 1).

Neodymium-doped yttrium aluminum garnet (Nd:YAG) laser	di Matteo et al. 2010 [35]	Swine pancreas	8	Laser treatment effect (coagulation necrosis) seen in ablated area in all gross specimens.	Peripancreatic fluid collection (*n* = 6) Elevated serum amylase (*n* = 7) Elevated serum lipase (*n* = 8).

High-intensity focused ultrasound (HIFU)	Hwang et al. 2009 [40]	Swine pancreas	12	HIFU treatment effect only seen at energy 1250 J (*n* = 2/4).	Adhesions of stomach and small intestine (*n* = 1), abdominal wall burns/injury (*n* = 3), gastric antral ulcer (*n* = 3), small bowel adhesions to pancreas (*n* = 2).

**Table 3:** EUS-guided ethanol ablation of pancreatic cysts (human studies).

References	Study design	*N*	Ethanol %	Outcome	Adverse events
Gan et al. 2005 [55]	Prospective single-center	25	5 to 80	23 patients with complete 6 month follow-up: complete resolution (*n* = 8), partial resolution (*n* = 2), persistent cyst (*n* = 8), surgical resection (*n* = 5).	Died of myocardial infarction 6 months after procedure (*n* = 1).

DeWitt et al. 2009 [56]	Prospective multicenter RCT	58	80	Single session ethanol lavage (*n* = 22) resulted in greater size pancreas cyst reduction (24%) compared to saline lavage (15%) (*n* = 17). 6/18 (33%) complete cyst resolution with ethanol lavage on follow-up CT imaging.	Abdominal pain (*n* = 8), acute pancreatitis (*n* = 1), major complications (*n* = 1).

Dimaio et al. 2011 [57]	Retrospective single-center	13	80	Mean max cyst diameter at baseline (20.1 ± 7.1 mm) decreased to 17.0 ± 9.8 mm (*P* = 0.06) after single ethanol lavage and to 12.8 ± 9.6 mm after two ethanol lavage sessions (*P* = 0.0002) Complete cyst resolution: after single session (0/13), after two sessions (5/13; 38%).	Minor abdominal pain after ethanol lavage (*n* = 1).

Oh et al. 2008 [59]	Prospective single-center	14	99% ethanol and 3 mg/mL paclitaxel (EUS-EPI)	Median original volume 3.81 (range 1.2–68 mL) decreased to 0.11 (range 0–35 mL) Complete cyst resolution (*n* = 11), partial resolution (*n* = 2), persistent (*n* = 1) at median 9 month follow-up.	Elevated serum amylase (*n* = 6) Mild pancreatitis (*n* = 1) Mild abdominal pain (*n* = 1).

Oh et al. 2011 [60]	Prospective single-center	52	EUS-EPI	85.8% reduction in cyst volume after EUS-EPI at 12 month follow-up. Complete resolution (*n* = 29), partial resolution (*n* = 6), persistent cyst (*n* = 12).	
